# Sacral Intradural Arachnoid Cyst of Cauda Equina: A Case Report and Review of the Literature

**DOI:** 10.7759/cureus.89673

**Published:** 2025-08-09

**Authors:** Mihail Kalnev, Atanas Davarski, Ivo Kehayov, Borislav Kitov

**Affiliations:** 1 Department of Neurosurgery, University Hospital Saint George, Plovdiv, BGR; 2 Department of Neurosurgery, Faculty of Medicine, Medical University of Plovdiv, Plovdiv, BGR

**Keywords:** chronic low back pain, intradural arachnoid cyst, polyradiculopathy, root compression, sacral cyst, tibial nerve

## Abstract

This report presents the case of a 36-year-old man complaining of chronic low back pain and numbness along the posterolateral surface of the right leg. Magnetic resonance imaging (MRI) revealed a disc degeneration and protrusion at the L_5_-S_1 _level and an extensive fluid-equivalent formation with a craniocaudal dimension of 8 cm at the S_1_-S_5 _level. Initially, due to the minimal clinical complaints, the cyst was considered asymptomatic. Two months later, the patient presented to the clinic with polyradiculopathy across S_1_-S_5 _dermatomes bilaterally, paresis of the tibial nerves, and retention of the pelvic-reservoir functions. Follow-up MRI confirmed the previous study, but visualized S_1_-S_5 _root compression. Given the presence of cauda equina syndrome, the patient was considered a surgical candidate. A cyst measuring 8 cm in craniocaudal length and 3 cm in width was found intradurally. Microsurgical resection of the cyst was performed. Histological examination confirmed the diagnosis of an arachnoid cyst. Postoperatively, due to recurrence, a lumbo-peritoneal shunt was implanted, after which the patient's neurological status significantly improved. Follow-up examination after 72 months revealed mild motor and sensory deficits. Maximal safe resection of the cyst is the most effective therapeutic approach that leads to the elimination of complaints and absence of recurrences. In cases with recurrence, a lumbo-peritoneal shunt may be considered.

## Introduction

Spinal arachnoid cysts (SACs) are rare lesions that account for nearly 1% of all spinal lesions [1]. The majority of SACs are usually asymptomatic, but in some cases, they can cause neurological deficits through compression of the medulla spinalis, cauda equina, or nerve roots [2]. According to their localization, SACs are extradural, intradural, extramedullary, or intramedullary [3]. Intradural cysts include arachnoid, enteric (endodermal, neuroenteric), and ependymal cysts [4].

Among all spinal canal cysts, arachnoid cysts are the most common, with intradural arachnoid cysts (IACs) accounting for 10% of all arachnoid cysts [5]. IACs can be seen anywhere along the spinal canal, with the literature suggesting that approximately 80% develop in the middle and lower thoracic region, 15% in the cervical region, and only 5-7% in the lumbar region [6,7].

This report presents a case of idiopathic IAC in the lumbo-sacral region with emphasis on the onset of clinical complaints, diagnostic and therapeutic options, disease outcome, and a review of the literature.

## Case presentation

A 36-year-old man presented with chronic low back pain for about two years, which gradually descended down to the posterolateral surface of the right leg. Magnetic resonance imaging (MRI) revealed disc degeneration at the level of L5-S1 and an arachnoid cyst in the sacral region with a craniocaudal dimension of 8 cm in the sagittal plane, starting from S1 to the upper surface of S5, more to the right. Initially, the cyst was assumed to be asymptomatic due to the minimal clinical complaints. Two months later, the patient developed low back pain, numbness, and weakness in both legs, and difficulty urinating.

**Figure 1:**
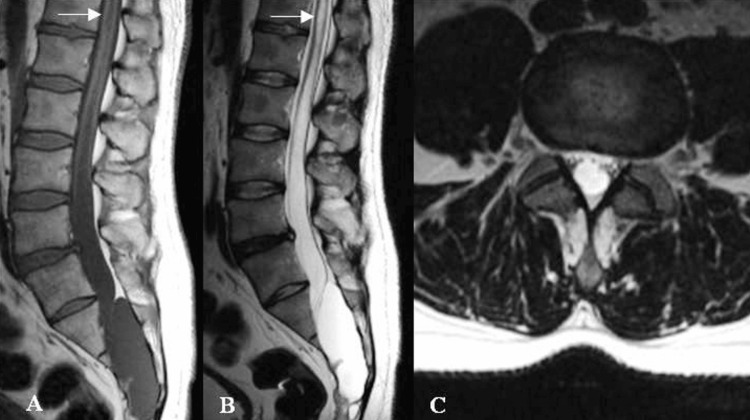
MRI of an intradural arachnoid cyst at the level of S1-S5. (A, B) Sagittal projections at T1 and T2 demonstrate cystic formation extending from the cranial surface of the S1 vertebra to S5 with liquor-signal intensity-hypointensity at T1 and hyperintensity at T2. (C) Axial projection shows the compressed nerve roots of the cauda equina.

The neurological status upon admission revealed low back pain with polyradiculopathy across S1-S5 dermatomes bilaterally, more on the right, weakened Achilles reflexes, paresis of the tibial nerves, and retention of pelvic reservoir functions. The neurological status was determined by the Modified McCormick scale of Grade III, and the disability grade of Grade 3 by the Modified Rankin Scale (mRS). Follow-up MRI confirmed the previous imaging study, with no increase in the size of the sacral intradural cyst. However, there was evidence of compression of the S1-S5 roots laterally and toward the ventral surface of the sacral canal (Figure 1).

Given the clinical presence of cauda equina syndrome, the patient was considered to be indicated for surgery. An intraoperative imaging guidance system, O-arm® Surgical Imaging System with StealthStation S7® navigation (Medtronic Sofamor Danek, Inc., Memphis, Tennessee, United States), was used to accurately determine the level of the lesion. Following sacrotomy, a tense dural sheath was visualized.

**Figure 2:**
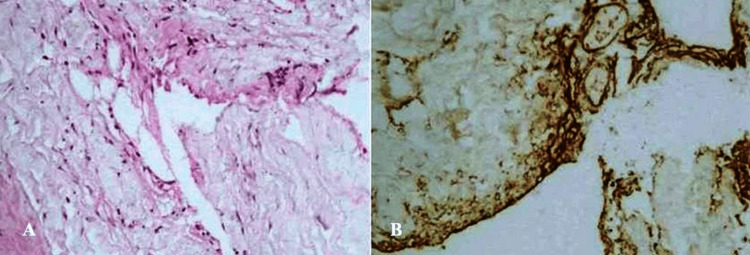
Intradural arachnoid cyst. (A) The cyst wall is represented by tender fibrous connective tissue, upholstered by meningothelial cells (H&E x 200); (B) Immunohistochemistry, EMA - positive expression in the cyst wall (x 400). H&E: hematoxylin and eosin; EMA: epithelial membrane antigen

After midline durotomy, an IAC measuring 8 cm in craniocaudal length and 3 cm in width was encountered. The latter was punctured by evacuating CSF under high pressure. A maximal safe resection of the IAC was attempted using meticulous microsurgical technique. A correction of the osteoclastic sacral defect was performed with a titanium mesh previously modelled (with Mesh Bender) to the defect, which was placed over the osteoclastic area and fixed circumferentially to the intact edges of the sacral bone with titanium screws. Histological examination of the cyst wall showed that it consisted of fibrocollagenous tissue, with meningothelial cells scattered among it (Figure 2).

**Figure 3:**
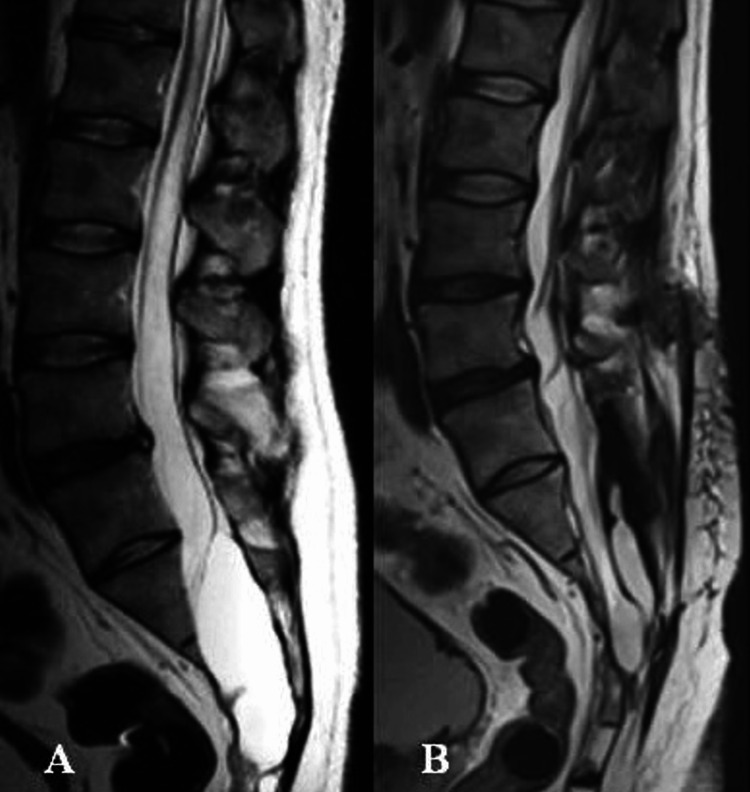
Comparisons of MRI of the patient with sacral intradural arachnoid cyst at S1-S5 level. (A) Preoperative; (B) Six years after surgery.

Postoperatively, due to the recurrence, it was necessary to perform a liquor drainage via a lumbo-peritoneal shunt, after which the patient's condition improved significantly; the tibial paresis decreased, and the urinary retention almost disappeared. At discharge, his neurological status was assessed at McCormick Grade II. At the 72-month follow-up, he was found to have mild motor and sensory deficits and was functionally independent. The degree of disability had improved from moderate (Grade 3) at discharge to minor (Grade 2), and at follow-up to Grade 1. An MRI performed six years after the surgical intervention showed effective decompression of cauda equina at the S1-S5 level with small residual cystic formations (Figure 3).

## Discussion

There is no consensus in the literature on the classification of SAC. The first classification was presented by Nabors et al. [8]. They divided SACs into three major types: (i) Type I, extradural cysts without nerve root fibers, (ii) Type II, extradural cysts containing nerve fibers, and (iii) Type III, intradural arachnoid cysts. Qi et al. divided SACs into five types: (i) Type 1, intramedullary cysts/syrinxes, (ii) Type 2, subdural extramedullary cysts, (iii) Type 3, subdural/epidural cysts, (iv) Type 4, intraspinal epidural cysts, and (v) type 5, intraspinal/extraspinal cysts [9]. The IAC in our patient corresponded to type III according to Nabors et al. [8] and type 2 according to Qi et al 9].

There are various theories in the literature attempting to explain the pathogenesis of SACs, which suggest that they arise from diverticula of the fibrous membrane connecting the arachnoid and pia mater along the posterior line of the spinal cord (septum posticum), diverticulation of the arachnoid layer in areas of decreased resistance, ectopic arachnoid granulations, chronic arachnoiditis, or abnormal proliferation of the arachnoid membrane creating valve mechanism between the subarachnoid space and the cyst, resulting in accumulation of liquor in the cystic cavity [2,10]. The existence of multiple pathogenetic theories, which have not been conclusively confirmed, indicates that a single etiological cause is unlikely to underlie the occurrence of SAC [11].

SACs can be congenital or acquired, for example, after trauma or intradural hematomas [12]. In the majority of cases, the causes of spinal IACs are not identified; therefore, they are considered congenital. In our case, it was assumed to be a congenital malformation, as there was no history of previous trauma or inflammatory diseases. This was supported by the presence of associated congenital malformations such as an intramedullary syrinx. A systematic review and meta-analysis performed by Kalsi et al. [13] found that all studies examining IAC, except one, that of Mohindra et al. [14], reported the presence of intramedullary syrinx. The data support the congenital nature of the cyst and its associated malformations. Congenital spinal IACs are usually asymptomatic and are diagnosed incidentally on imaging because of complaints caused by another disease [15], a fact which is confirmed by our case. 

It has been documented that congenital asymptomatic IACs can increase in volume and become symptomatic, both due to trauma and as a consequence of the increase in intra-abdominal and intra-thoracic pressure [4,6]. When intra-abdominal and intrathoracic pressures increase, CSF enters during systolic pulsation but cannot exit through the same "portal" during diastole due to the effect of a one-way valve on the cyst neck, which gradually increases its size [16]. Other mechanisms of IAC growth include active secretion from cells covering the inner layer of the cyst, osmotic penetration of CSF from the subarachnoid space, and a change in CSF pulsation dynamics [1]

The incidence of IACs of cauda equina ranges from 5-10% of all spinal cysts [7,13,16]. Our findings of an intradural arachnoid cyst localized in the cauda equina represent < 0.1 per 100,000 hospitalized patients, 0.25% of all spinal tumors, 1.6% of IETs, and 5.3% of intradural lesions in the cauda equina, as per our hospital records.

The gender distribution of patients with intradural extramedullary arachnoid cysts varies widely in the literature, from a female-to-male ratio of 3.4:1 to 1:2.7, suggesting that gender is not significant [11,13]. IACs occur most frequently between the third and fourth decades of life, and the mean age of patients, in most published series, ranges from 35 to 55 years [1,17], which is identical to that of our patient, a 36-year-old man.

Clinical symptoms in patients with IAC develop over months, with one-third of them fluctuating in nature [1]. The clinical picture develops depending on the localization of the cyst and usually presents with low back pain, motor and sphincter disturbances, while sensory deficits are not pronounced [1]. 

Spondylography is not particularly useful in the diagnosis of spinal IAC, but in some cases, it can visualize dorsal canal enlargement as well as pedicle thinning and increased interpedicular distance due to longstanding cyst compression [13]. Similar to spondylography, computed tomography (CT) is also able to visualize indirect evidence of bony erosion and spinal canal enlargement. Until the mass introduction of MRI, myelography and CT-assisted myelography were the techniques of choice in the diagnosis of IAC. Myelography and CT-assisted myelography were able to detect a "stop" or defect in the filling of the contrast material, the communication of the subarachnoid space with the cyst, and to refine the degree of free flow of the CSF between the subarachnoid space and the cyst and the possible rupture of the dura mater [18]. Currently, these examinations are rarely used as they are invasive and with significant X-ray exposure. Performing a lumbar puncture to inject the contrast material carries the risk of damaging the conus medullaris in cases where the latter is inferiorly ending, as in our patient. Although these examinations are historical, they can still play an important diagnostic role when, on performing an MRI, the diagnosis of IAC is not definite or when an MRI examination is contraindicated [13,17]

Nowadays, MRI represents the gold standard in the diagnosis of IAC [1,19]. MRI is able to accurately determine both the localization and size of the cyst, as well as the spinal cord changes present, such as low-lying conus medullaris, presence of syrinx, and atrophy [19]. The imaging characteristics are identical to those of CSF. The signal is hypointense in T1 and hyperintense in T2. When contrast material is injected, it does not drip into the cyst [20].

Surgical intervention is considered the main treatment for symptomatic SACs [2]. The goal of surgical treatment is to decompress the compressed nerve structures and prevent new filling of the cyst by its complete resection and closing the communication between it and the subarachnoid space, leading to the elimination of complaints [6]. Unfortunately, total excision of the cyst is not always possible, so surgical intervention often involves decompression, fenestration, and maximal safe resection of the cyst. In cases of recurrence, a cysto-peritoneal shunt may be considered [1,21], which we performed in our patient. Aspiration of the cyst contents alone is not recommended because of the high recurrence rate, whereas after complete excision of the cyst, with tight closure of the communication, very good results are obtained and recurrences are rare [22]. The prognosis is worse when total excision of the cyst is not achieved or the communication cannot be interrupted [2]

In a long-term follow-up (mean eight years) of 34 patients operated on for SACs, El-Hajj et al. found that sensory neuropathy was the symptom that most often improved (81%), followed by pain (74%) and motor disturbances (64%) [7]. Another study found improvement in motor function in 71% of patients, sensory neuropathy in 64% and pain in 50% [17]. 

The follow-up examination of our patient 72 months after the intervention showed that the low back pain, although reduced, persisted, allowing him to take care of himself unassisted, but limiting his social life. In smaller series of patients with SAC treated surgically, complication rates have been reported to range from 0% to 27% [7,13,23].

## Conclusions

SACs are a rare but treatable pathology. Decompression of the compressed nerve structures by total excision of the cyst and closure of the communication with the subarachnoid space is the most effective therapeutic approach, which leads to the elimination of complaints and absence of recurrences. In cases of recurrence, it is necessary to consider performing a cysto-peritoneal shunt with close follow-up of the patient.
